# Comparative Evaluation of Antibacterial Activity of Three Universal Bonding Agents Against Streptococcus mutans on Demineralized Dentin: An In Vitro Study

**DOI:** 10.7759/cureus.103720

**Published:** 2026-02-16

**Authors:** Kirti Rathee, Charu Dayal, Reena Rani, A Anukriti, Anjum Zia, Ankita Sundan, Seema Gupta

**Affiliations:** 1 Department of Conservative Dentistry and Endodontics, Institute of Dental Studies and Technologies, Ghaziabad, IND; 2 Department of Pedodontics and Preventive Dentistry, Santosh Dental College and Hospital, Ghaziabad, IND; 3 Department of Conservative Dentistry and Endodontics, Inderprastha Dental College and Hospital, Ghaziabad, IND; 4 Department of Pedodontics and Preventive Dentistry, Zia’s Family Dental Care, Baramulla, IND; 5 Department of Pedodontics and Preventive Dentistry, Dr. Neelam Shourie’s City Dental Clinic, Panchkula, IND; 6 Department of Orthodontics, Kothiwal Dental College and Research Centre, Moradabad, IND

**Keywords:** antibacterial, bonding agents, dentin, dentistry, restorative, streptococcus mutans

## Abstract

Introduction: Secondary caries remains a leading cause of restoration failure, often resulting from residual bacteria persisting in demineralized dentin after minimally invasive cavity preparation. The incorporation of inherent antibacterial properties into universal bonding agents might offer a promising strategy to reduce this risk without additional disinfection steps. The aim of this in vitro study was to comparatively evaluate the antibacterial activity of three universal bonding agents against *Streptococcus mutans* using demineralized human dentin blocks as a simulated carious dentin model.

Materials and methods: A total of 150 dentin blocks (4 mm × 4 mm, ~200 µm thick) were prepared from extracted human permanent molars, sterilized, and demineralized in 0.2 M sodium acetate buffer (pH 3.0) for four weeks, followed by washing in 0.02 M phosphate-buffered saline for one week. Each block was inoculated with 0.2 µL of *S. mutans* suspension and allowed to adsorb for 10 min. The blocks were randomly divided into five groups (n = 30 each): One Coat 7.0 Universal (Coltène/Whaledent AG, Altstätten, Switzerland), Single Bond Universal (3M ESPE/Solventum, St. Paul, Minnesota, USA), Tetric N-Bond Universal (Ivoclar Vivadent AG, Schaan, Liechtenstein), 0.2% chlorhexidine (positive control), and normal saline (negative control). Bonding agents were applied for 20 s in self-etch mode without light curing. Treated blocks were fragmented, homogenized, serially diluted, and plated on Mueller-Hinton agar. Plates were incubated anaerobically at 37 °C for 48 h, and colony-forming units (CFUs) were counted. Log-transformed CFU data were analyzed using one-way analysis of variance, followed by Tukey’s honestly significant difference post hoc test (α = 0.05).

Results: A statistically significant difference was observed between groups (F = 425.5, p = 0.001). The positive control (0.2% chlorhexidine) demonstrated the greatest bacterial reduction, followed by the universal bonding agents, which were significantly superior to that of the negative control (p = 0.001). Among the adhesives, Tetric N-Bond Universal exhibited significantly stronger antibacterial activity than One Coat 7.0 Universal (p = 0.024), whereas Single Bond Universal showed intermediate efficacy. All bonding agents achieved substantial CFU reduction relative to saline but remained inferior to chlorhexidine.

Conclusion: Universal bonding agents possess inherent antibacterial properties against *S. mutans* on demineralized dentin, with efficacy varying according to formulation and pH profile. Although not as potent as chlorhexidine, these agents may contribute to residual bacterial control in minimally invasive restorations, potentially lowering the risk of secondary caries. Long-term in vivo studies are required to confirm their clinical relevance.

## Introduction

Modern restorative dentistry emphasizes the preservation of healthy tooth structures through minimally invasive approaches, marking a significant shift from traditional principles such as G.V. Black’s “extension for prevention” to the contemporary concept of minimally invasive dentistry [1,2]. This paradigm prioritizes conservative cavity preparation to maintain the maximum natural tooth tissue. However, despite careful removal of infected dentin, residual bacteria often persist within the smear layer, dentinal tubules, or demineralized affected dentin, particularly in deep carious lesions, where complete excavation may risk pulpal exposure [2,3].

These residual microorganisms significantly contribute to secondary caries, accounting for approximately 60% of restoration failures and replacements. Secondary caries typically arises from microleakage at the tooth-restoration interface, allowing bacterial penetration, proliferation, and subsequent pulpal inflammation [4]. With the growing demand for esthetic composite restorations, the management of microleakage and bacterial contamination remains a persistent clinical challenge.

Conventional cavity disinfection methods, such as acid etching with phosphoric acid followed by rinsing, provide only limited bacterial reduction and are not reliably effective against residual pathogens [5]. Consequently, there is increasing interest in restorative materials and adhesive systems that exhibit inherent antibacterial properties to eliminate or inhibit surviving cariogenic bacteria, thereby improving the long-term success of minimally invasive restorations and reducing the incidence of secondary caries.

Contemporary universal bonding agents, designed for versatility (self-etch, selective-etch, or total-etch modes), incorporate acidic monomers such as 10-methacryloyloxydecyl dihydrogen phosphate (MDP) and exhibit low pH values that may contribute to antibacterial effects [6]. These properties make them promising candidates for reducing bacterial colonization at the dentin-restoration interface.

*Streptococcus mutans*, a primary etiological agent in dental caries initiation and progression, serves as a representative cariogenic bacterium for evaluating antimicrobial efficacy because of its role in biofilm formation and acid production [7]. The present in vitro study was therefore conducted to comparatively evaluate the antibacterial activity of three commercially available universal bonding agents such as One Coat 7.0 Universal (Coltene), Single Bond Universal (3M ESPE), and Tetric N-Bond Universal (Ivoclar Vivadent), against *Streptococcus mutans* using demineralized dentin blocks inoculated with the bacterium. The objectives of this in vitro study were to: (1) quantify the reduction in colony-forming units (CFUs) achieved by each bonding agent; (2) compare their efficacy relative to 0.2% chlorhexidine (positive control) and normal saline (negative control); and (3) determine whether any of the tested agents demonstrated statistically significant antibacterial potential suitable for reducing the risk of secondary caries in minimally invasive restorations.

## Materials and methods

Study design

This in vitro experimental study was conducted in the Department of Conservative Dentistry and Endodontics at the Inderprastha Dental College, Ghaziabad, from June 2025 to December 2025. Ethical approval was obtained from the Institutional Ethical Committee (EC/NEW/INST/2024/UP/0230), and informed consent was obtained from patients to use their teeth for study purposes.

Materials employed

The bacterial strain used was *Streptococcus mutans* MTCC 497 (IMTECH, Chandigarh). Demineralization was performed using 0.2 M sodium acetate buffer at pH 3.0, followed by washing in 0.02 M phosphate buffered saline (PBS). Culture media consisted of Brain Heart Infusion (BHI) broth and Mueller-Hinton agar (MHA) plates. The use of MHA in this study is justified because the experiment involved a standardized pure culture of Streptococcus mutans under controlled in vitro conditions, eliminating the need for selective or enriched media. MHA is the Clinical and Laboratory Standards Institute (CLSI)-recommended standard medium for antimicrobial testing, offering reproducible composition, minimal inhibitory substances, and reliable bacterial recovery. Since the objective was quantitative CFU enumeration after exposure to bonding agents, a non-selective medium ensures unbiased viability assessment. The positive control was 0.2% chlorhexidine gluconate, whereas normal saline served as the negative control. The universal bonding agents tested included one-coat 7.0 universal (Coltene/Whaledent AG, Altstätten, Switzerland), Single Bond Universal (3M ESPE, 3M Company, St. Paul, Minnesota, USA), and Tetric N-Bond Universal (Ivoclar Vivadent AG, Schaan, Liechtenstein). Additional materials included silicon carbide abrasive paper, distilled water, a Biological Oxygen Demand (BOD) incubator, and an autoclave for sterilization.

Sample preparation

Freshly extracted, intact human permanent molars were collected, cleaned, washed, and stored in distilled water at room temperature. Dentin blocks with dimensions of 4 mm × 4 mm (approximately 200 µm thickness after polishing with silicone carbide abrasive paper) were prepared under water cooling using a diamond disc. Dentin blocks were sterilized in a steam autoclave at 121 °C under 15 psi pressure for 30 minutes, and sterility was confirmed by incubating representative samples on MHA plates that showed no growth.

Demineralization and bacterial inoculation

Dentin blocks were immersed in 0.2 M sodium acetate buffer (pH 3.0) for four weeks, with weekly buffer changes, to simulate demineralized carious dentin. Blocks were then washed in 0.02 M PBS for one week. A standardized 0.2 µL aliquot of *S. mutans* suspension in PBS was applied to each block surface and allowed to adsorb for 10 min to enable bacterial colonization and penetration into the dentinal tubules.

Grouping and application of test agents

The inoculated blocks were randomly divided into five groups (n = 30 each): Group A received One Coat 7.0 Universal (Coltene), Group B received Single Bond Universal (3M ESPE), Group C received Tetric N-Bond Universal (Ivoclar Vivadent), Group D received 0.2% chlorhexidine (positive control), and Group E received normal saline (negative control). Each agent or control was applied for 20 s per manufacturer’s instructions in self-etch mode, without prior etching or light curing, to assess the immediate antibacterial effects of unpolymerized components.

Bacterial recovery and viability assessment

The treated blocks were fragmented and homogenized in 500 µL of the respective solutions. The homogenate was serially diluted in BHI broth, and 100 µL aliquots were spread-plated onto MHA plates in triplicate. The plates were incubated anaerobically at 37 °C for 48 h in a BOD incubator. CFU were counted and expressed per block or per mL homogenate. Each sample was plated in triplicate to ensure technical reliability. The three CFU counts obtained from plates corresponding to the same dentin block were averaged to generate a single mean CFU value per block. These averaged values were used for subsequent statistical analysis.

Sample size calculation

The sample size was determined a priori using the G*Power software (version 3.1.9, Heinrich-Heine-Universität Düsseldorf). Based on an analysis of variance (ANOVA) model for multi-group comparisons, a minimum sample of 150 dentin blocks (30 per group) was required to detect an effect size of 0.28 (derived from prior studies on the antimicrobial efficacy of bonding agents) with 80% power and a 5% alpha error [8].

Statistical analysis

Statistical analysis was performed using IBM SPSS Statistics for Windows, Version 23 (Released 2015; IBM Corp., Armonk, New York, United States). The CFU data were log-transformed prior to analysis. Normality of the transformed data for each of the five groups was confirmed using the Shapiro-Wilk test. One-way ANOVA was conducted to test for overall differences between group means at an alpha level of 0.05, followed by Tukey’s honestly significant difference test for post hoc pairwise comparisons.

## Results

One-way ANOVA demonstrated a statistically significant difference in the mean CFUs (log-transformed CFUs) across the five treatment groups (p = 0.001), as presented in Table 1. 0.2% chlorhexidine demonstrated the strongest antibacterial effect (mean = 5.3 ± 2.1 CFU), while normal saline showed the weakest (155.6 ± 15.3 CFU). The three universal bonding agents (Groups A, B, and C) exhibited moderate and intermediate efficacy, with Tetric N-Bond Universal showing the lowest mean CFU count. These results confirm that although the bonding agents possess inherent antibacterial properties, their efficacy is significantly lower than that of the 0.2% chlorhexidine positive control.

**Table 1:** Comparison of colony-forming units (CFUs) of S. mutans between dentin bonding agents with the one-way analysis of variance (ANOVA) test. *Statistically significant (p < 0.05). Values are presented as mean and standard deviation (SD), CFUs: colony-forming units; df: degree of freedom. Descriptive statistics are presented as raw CFU counts (mean ± SD, median, min–max), whereas one-way ANOVA and Tukey’s HSD comparisons were performed on log-transformed CFU values.

Group (Treatment)	N	Mean ± SD (CFU)	Median (CFU)	Min-Max (CFU)	Range	F statistics	df	p-value
A: One Coat 7.0 Universal	30	42.5 ± 8.2	41.0	30-58	28	425.5	4	0.001*
B: Single Bond Universal	30	38.2 ± 7.5	37.0	26-52	26			
C: Tetric N-Bond Universal	30	35.8 ± 6.9	35.0	21-52	31			
D: 0.2% Chlorhexidine (Positive control)	30	5.3 ± 2.1	5.0	2-10	8			
E: Normal Saline (Negative control)	30	155.6 ± 15.3	152.0	130-185	55			

The post hoc Tukey test (Table 2) established a hierarchy of efficacy. All bonding agents were significantly less effective than chlorhexidine (p = 0.001) but significantly better than saline (p = 0.001). Among the adhesives, Tetric N-Bond Universal showed a significantly stronger antibacterial activity than One Coat 7.0 Universal (mean difference = 6.70 CFU, p = 0.024). No significant differences were found between Single Bond Universal and the other two bonding agents (p > 0.05). These findings indicate that, while universal bonding agents possess intrinsic antibacterial properties against *S. mutans*, their efficacy remains limited compared to chlorhexidine, with Tetric N-Bond Universal demonstrating the most promising performance among the tested adhesives.

**Table 2:** Post hoc pairwise comparisons (Tukey's HSD test) of mean differences in log-transformed colony-forming units (CFUs) between treatment groups. *Statistically significant (p < 0.05)

Comparison Pairwise	Mean Difference (CFU)	t-statistic	p-value	95% Confidence Interval (CFU)
One Coat 7.0 vs. Single Bond	4.30	1.92	0.277	(-1.92, 10.52)
One Coat 7.0 vs. Tetric N-Bond	6.70	2.99	0.024*	(0.48, 12.92)
One Coat 7.0 vs. Chlorhexidine	37.20	16.61	0.001*	(30.98, 43.42)
One Coat 7.0 vs. Saline	-113.10	-50.52	0.001*	(-119.32, -106.88)
Single Bond vs. Tetric N-Bond	2.40	1.07	0.789	(-3.82, 8.62)
Single Bond vs. Chlorhexidine	32.90	14.69	0.001*	(26.68, 39.12)
Single Bond vs. Saline	-117.40	-52.44	0.001*	(-123.62, -111.18)
Tetric N-Bond vs. Chlorhexidine	30.50	13.62	0.001*	(24.28, 36.72)
Tetric N-Bond vs. Saline	-119.80	-53.51	0.001*	(-126.02, -113.58)
Chlorhexidine vs. Saline	-150.30	-67.13	0.001*	(-156.52, -144.08)

## Discussion

This study evaluated the immediate antibacterial effect of the bonding agents on their unpolymerized (pre-curing) state, thereby capturing the direct antimicrobial influence of acidic monomers and other reactive components. However, in clinical practice, adhesives are polymerized immediately after application, which significantly reduces monomer availability and may diminish antibacterial activity. Therefore, the present findings reflect the initial chemical antibacterial potential rather than the sustained post-polymerization effect at the tooth-restoration interface. The findings of this in vitro study underscore the varying antibacterial potential of universal bonding agents against *S. mutans*, a key cariogenic pathogen implicated in secondary caries development. The superior performance of chlorhexidine aligns with its established mechanism as a broad-spectrum antimicrobial agent that disrupts bacterial cell membranes and coagulates intracellular proteins, effectively reducing viable bacterial counts in demineralized dentin models. This reaffirms its role as a benchmark for cavity disinfection, consistent with prior research emphasizing its efficacy in preventing residual bacterial proliferation at the tooth-restoration interface [9].

Among the tested universal bonding agents, the observed hierarchy in antibacterial activity, with Tetric N-Bond Universal demonstrating significantly stronger inhibition than One Coat 7.0 Universal, whereas Single Bond Universal showed intermediate effects, which can be attributed to differences in their chemical compositions and pH profiles. Universal adhesives typically incorporate acidic monomers such as MDP, which contribute to antibacterial effects through low pH-induced bacterial membrane disruption and potential chelation with calcium ions in dentin, forming salts that neutralize acidity over time [6,10]. Tetric N-Bond Universal with the mildest pH (approximately 2.5-3.0) exhibited the most pronounced antibacterial effect among the adhesives, possibly due to optimized monomer concentrations or additional stabilizers that enhance sustained interactions with bacterial cells without rapid buffering by dentin [11].

In contrast, One Coat 7.0 Universal with a lower pH (approximately 2.0) showed comparatively weaker activity, which may result from quicker neutralization upon dentin contact, diminishing its bactericidal potency as the acidic monomers are expended. A previous study reported similar results with Tetric N-Bond, demonstrating superior antimicrobial activity than One Coat 7.0 universal [12]. Single Bond Universal, with a pH of approximately 2.7, likely balances these factors by incorporating copolymers and fillers that provide moderate antibacterial support. These variations highlight how formulation specifics, beyond mere acidity, influence efficacy. For instance, the presence of hydrophilic components such as 2-hydroxyethyl methacrylate or silane in some agents may facilitate better penetration into dentinal tubules, targeting intratubular bacteria more effectively [13,14].

Support for these interpretations was drawn from previous investigations of self-etch adhesives. Imazato et al. [15] demonstrated that unpolymerized acidic monomers in dentin primers exert antibacterial effects against *S. mutans* in carious dentin, attributing this to low pH and quaternary ammonium compounds, such as methacryloyloxydodecylpyridinium bromide (MDPB), although the latter was not present here. Kim and Shin [8] evaluated self-etch adhesives against *S. mutans *and found that pH alone does not dictate efficacy. Instead, monomer hydrophilicity and ethanol content enhance bacterial inhibition by improving wettability and diffusion. Their results corroborate the intermediate effects observed in this study, where ethanol-based formulations likely aided bacterial contact. Furthermore, a previous study reported that the antimicrobial activity of dentin bonding agents is pH dependent, with multiple bonding systems showing significant differences in CFU reduction [16]. Ahmed et al. [17] linked residual bacteria to restoration failures, supporting the need for adhesives with inherent antibacterial properties to mitigate secondary caries, which account for 60% of replacements.

These outcomes also resonate with those of studies on bacterial colonization in minimally invasive preparations. A narrative review by Alyahya [18] advocated for antibacterial materials in conservative dentistry to preserve tooth structure, whereas Browne and Tobias [19] highlighted microleakage as a pathway for pulpal inflammation. The demineralized dentin model used here simulates clinical scenarios where the affected dentin harbors viable bacteria, as described by Imazato et al. [20], who showed that primers with MDPB effectively reduced bacterial viability in human carious dentin.

In terms of clinical implications, these results suggest that selecting universal bonding agents with optimized antibacterial profiles could enhance the longevity of composite restorations in minimally invasive procedures, particularly in high-risk patients, by reducing residual *S. mutans* and minimizing the risk of secondary caries without additional disinfectants. However, clinicians should integrate these techniques with appropriate etching techniques for optimal bonding. The present study has several limitations that should be considered when interpreting the findings. As an in vitro investigation, it does not fully replicate the complexity of the oral environment, including multispecies biofilm dynamics, salivary buffering and protein interactions, host immune responses, thermal fluctuations, and mechanical stresses. The use of a mono-species *Streptococcus mutans* model and controlled laboratory conditions may not accurately reflect bacterial behavior within mature polymicrobial biofilms in vivo. Additionally, the study evaluated only the immediate antibacterial activity under uncured/self-etch conditions, without assessing post-polymerization effects, aging, degradation, or long-term antibacterial performance. These factors may significantly influence clinical outcomes and limit the direct generalizability of the results. Therefore, the findings should be interpreted as evidence of initial antibacterial potential rather than sustained clinical efficacy.

## Conclusions

This in vitro study demonstrated that universal bonding agents possess inherent antibacterial activity against *S. mutans* on demineralized dentin blocks, with Tetric N-Bond Universal exhibiting significantly greater bacterial reduction than One Coat 7.0 Universal, while Single Bond Universal showed intermediate efficacy. Although all three agents achieved substantial CFU reduction, none matched the potent antimicrobial effect of the 0.2% chlorhexidine. These findings highlight the potential of universal adhesives to contribute to residual bacterial control in minimally invasive restorations, thereby potentially reducing the risk of secondary caries. However, their antibacterial effects remain limited and formulation-dependent. While universal bonding agents demonstrate inherent immediate antibacterial activity against *S. mutans*, these results represent pre-curing conditions and may not fully translate to clinical post-polymerization scenarios. Further studies evaluating antibacterial effects after light curing and long-term aging are warranted. 
